# Higher Expression of Toll-Like Receptors 2 and 4 in Blood Cells of Keratoconus Patiens

**DOI:** 10.1038/s41598-017-13525-7

**Published:** 2017-10-11

**Authors:** Tomás Sobrino, Uxía Regueiro, Mercedes Malfeito, Alba Vieites-Prado, María Pérez-Mato, Francisco Campos, Isabel Lema

**Affiliations:** 1 Clinical Neurosciences Research Laboratory, Hospital Clínico Universitario, Universidade de Santiago de Compostela, Health Research Institute of Santiago de Compostela (IDIS), Santiago de Compostela, Spain; 2 Facultade de Óptica e Optometría, Universidade de Santiago de Compostela, Santiago de Compostela, Spain; 3 Instituto Galego de Oftalmoloxía (INGO), Hospital Provincial de Conxo, Santiago de Compostela, Spain

## Abstract

Inflammation may play a significant role in Keratoconus (KC), but the implication of immunity on this inflammatory response is unknown. Therefore, our aim was to determine the expression levels of Toll-like receptors 2 (TLR2) and 4 (TLR4) in monocytes and neutrophils from patients with KC and control subjects for demonstrating the role of innate immunity in KC. We also study the correlation between TLR2/TLR4 expression and serum levels of proinflammatory markers (IL-1β, IL-6, TNF-α, MMP-9 and NF-κB). Forty patients with bilateral KC (55% males; mean age; 33.1 ± 10.9 years) and 20 control subjects (55% males; mean age; 30.4 ± 7.6 years) were included in the study. Our results showed that mean expression of TLR2 and TLR4 in both neutrophils and monocytes was significantly higher in patients with KC compared to control subjects (all p < 0.0001). Furthermore, KC patients also showed higher serum levels of IL-1B, IL-6, TNF-α, MMP-9 (all p < 0.0001) and NF-κB (p = 0.036). In addition, we found a strong correlation between TLR2 expression in both monocytes and neutrophils (all p < 0.0001), and TLR4 in monocytes (all p < 0.05) with serum levels of IL-1B, IL-6, TNF-α and MMP-9. In conclusion, these findings suggest that TLRs may play an important role in the pathophysiology of KC.

## Introduction

Keratoconus (KC) is a primary corneal ectasia, generally bilateral and progressive, in which the cornea assumes a conical shape as a result of the thinning of the corneal stroma. The corneal thinning induces irregular astigmatism, myopia and protrusion, leading to mild to marked impairment in the quality of vision^1^. On the other hand, the cornea is part of an integrated system, the ocular surface, which contains specific and non-specific immune molecules. In this regard, tissue degradation in thinning disorders as KC involves the expression of inflammatory mediators, such as proinflammatory cytokines, cell adhesion molecules and matrix metalloproteinases^2–4^. Moreover, previous studies by our group have demonstrated that levels of pro-inflammatory cytokines, such as IL-6 and TNF-α, and matrix metalloproteinase-9 (MMP-9) are significantly increased in tears of patients with KC^4,5^. We also observed decreased levels of lactoferrin (Lf), zinc-α2-glycoprotein (ZAG), and immunoglobulin kappa chain (IGKC) in the tears of patients with KC compared to control subjects^6^. All these molecules are involved in the immune response, so these results suggest that immunological processes may be involved in the pathogenesis of ectasias. In fact, Lf, at systemic and local level, modulates the function of humoral and cellular components of the innate and adaptive response via toll like receptors (TLRs)^7,8^. TLRs are a family of highly conserved innate immunity receptors that recognize damage-associated molecular patterns from endogenous molecules, which are released as a consequence of tissue damage^9^. These molecules, also known as endogenous ligands, are able to activate TLR promoting the recruitment of several adaptive proteins to activate nuclear factor-kB (NF-κB), which induces the expression of pro-inflammatory genes, inflammatory cytokines, and adhesion molecules^10^. In this regard, previous studies by our group have demonstrated that inflammatory markers are associated with KC and subclinical KC^4,5^. Moreover, TLR4 is expressed in several eye tissues and cells, including corneal epithelial cells and fibroblasts of the corneal stroma. TLR2 is also expressed in human conjunctival epithelial cells^8^. Importantly, the treatment of corneal epithelial cells with neutralizing TLR2-antibodies induced a significant decrease of IL-6, IL-8 and TNF-α^11^. Consequently, it is tempting to postulate that the inflammatory response associated to KC could be a consequence of the TLR activation. However, the role of TLRs in KC patients has not yet been tested. Therefore, the purpose of this study was to measure expression levels of TLR2 and TLR4 in monocytes and neutrophils from KC patients and control subjects. In addition, we study the correlation between TLR2/TLR4 expression and serum levels of proinflammatory markers (IL-1B, IL-6, TNF-α, MMP-9 and NF-κB).

## Results

We prospectively studied 40 patients with bilateral KC (55% male; mean age, 31.1 ± 10.9 years) and 20 control subjects (55% male; mean age, 30.4 ± 7.6 years). No age- and sex-related statistical differences were detected between patients with KC and control subjects. However, patients with KC showed more frequent family history of KC and allergic disease (Table 1). Twenty-four KC patients (60%) and 6 control subjects (30.0%) reported allergic disease; and 10 KC patients (25%) but no control subjects a family history of KC.

**Table 1:** Clinical characteristics, topographic variables, biochemical parameters and TLR expression in patients with keratoconus and control subjects.

	**Controls n = 20**	**Keratoconus n = 40**	**p**
Age (years)	30.4 ± 7.6	33.1 ± 10.9	0.276
Male (%)	55	55	0.609
Allergic disease (%)	30	60	0.028
Family history of KC	0	25	0.001
K_2_ (diopters)	43.4 ± 1.2	54.3 ± 5.9	<0.0001
Thinnest point (μm)	534.2 ± 32.1	410.5 ± 61.1	<0.0001
TLR2 neutrophils (AFU)	539.2 ± 114.8	718.8 ± 92.3	<0.0001
TLR2 monocytes (AFU)	2222.5 ± 415.8	2837.6 ± 487.1	<0.0001
TLR4 neutrophils (AFU)	480.1 ± 129.8	678.9 ± 116.2	<0.0001
TLR4 monocytes (AFU)	1033.6 ± 111.4	1613.4 ± 277.8	<0.0001
IL-1β (pg/mL)	63.0 ± 38.1	123.9 ± 53.8	<0.0001
IL-6 (pg/mL)	75.9 ± 41.6	112.4 ± 29.6	<0.0001
TNF-α (pg/mL)	35.9 ± 24.5	93.2 ± 59.0	<0.0001
MMP-9 (ng/mL)	16.8 ± 3.8	25.9 ± 6.4	<0.0001
NF-κB (∆Ct)	5.9 ± 4.8	3.0 ± 4.4	0.036

IL = interleukin; K_2_ = steep meridian; KC = keratoconus; MMP-9 = matrix metalloproteinase 9; NF-κB = nuclear factor-kappa B; TNF-α = tumor necrosis factor alpha; TLR = Toll-like receptor.

The elapsed time from the first diagnosis of KC eye and the study ranged from 1 to 31 years (mean, 12.1 ± 9.7 years). All KC patients showed moderate or severe stage of KC progression in the most affected eye: 17 eyes (42.5%) presented moderate KC (K_2_ between 45 D and 52 D); and 23 eyes (57.5%) had severe KC (K_2_ > 52 D). Moreover, Table 1 also shows the mean K_2_ and thinnest point values for the study groups. Mean K_2_ values were higher and mean thinnest point lower in the eyes with KC *versus* the control ones (p < 0.0001).

### Expression of TLR2/TLR4 and proinflammatory molecules

**Figure 1:**
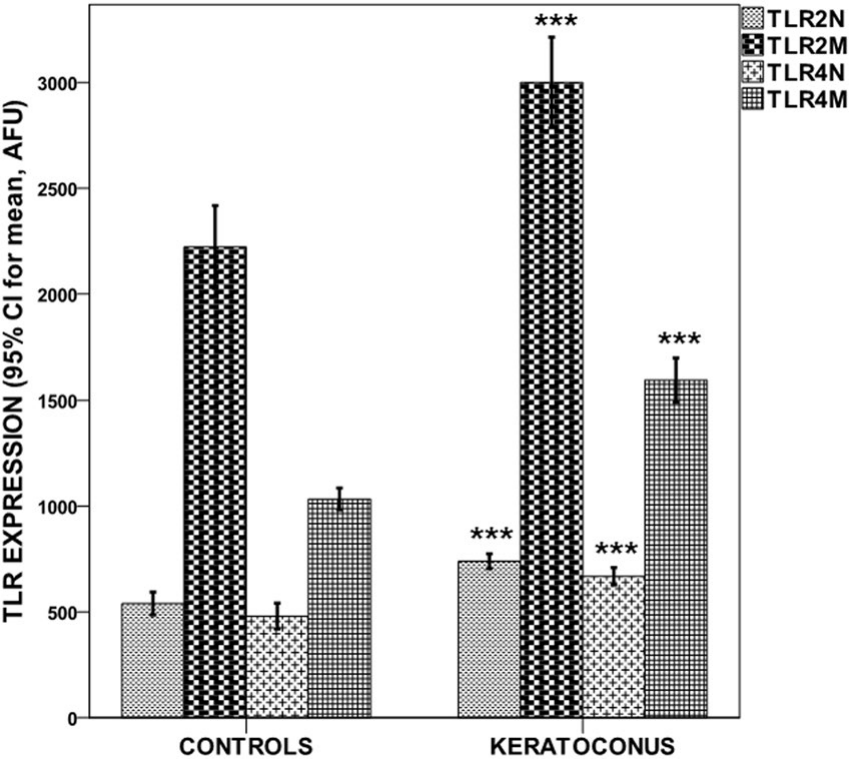
TLR2/TLR4 expression levels in neutrophils/monocytes by study groups. Values represent the 95% CI for mean. ***p < 0.0001 compared to controls.

**Figure 2:**
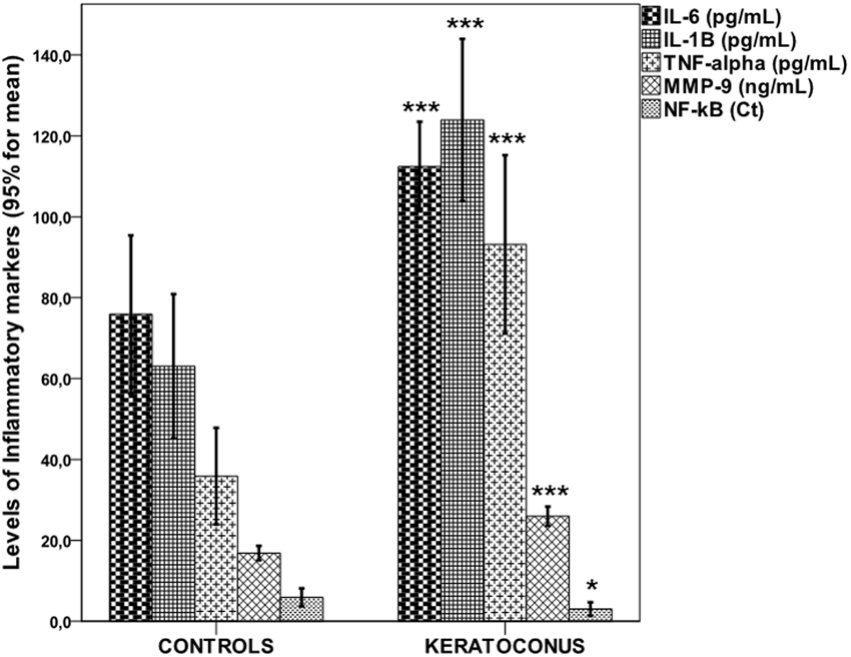
Expression levels of inflammatory markers by study groups. Values represent the 95% CI for mean. *p < 0.05; ***p < 0.0001 compared to controls.

Figure 1 shows that the mean expression of TLR2 and TLR4 in both neutrophils and monocytes was significantly higher in patients with KC compared to control subjects (all p < 0.0001) (Table 1). Moreover, KC patients had higher serum levels of IL-1B, IL-6, TNF-α, MMP-9 (all p < 0.0001) and NF-κB (p = 0.036) (Fig. 2) (Table 1). On the other hand, no TLR expression differences were observed between KC patients with and without allergic disease (TLR2 expression in neutrophils (759.1 ± 108.8 vs. 709.7 ± 108.3; p = 0.167); TLR2 expression in monocytes (3139.3 ± 715.5 vs. 2790.9 ± 557.8; p = 0.109); TLR4 expression in neutrophils (664.0 ± 120.4 vs. 674.8 ± 144.1; p = 0.800); TLR4 expression in monocytes (1574.8 ± 308.5 vs. 1621.7 ± 352.8; p = 0.659).

### Correlation between the expression of TLR2/TLR4 and severity of KC

No association was found between the expression of TLR2 and TLR4 in both neutrophils and monocytes and the severity of the most affected eye with KC, evaluated by the steep keratometric reading (K_2_) and the thinnest point of the cornea.

Pearson coefficients for K_2_ were the following: TLR2 expression in neutrophils (r = 0.041; p = 0.800); TLR2 expression in monocytes (r = −0.048; p = 0.767); TLR4 expression in neutrophils (r = 0.187; p = 0.249); and TLR4 expression in monocytes (r = 0.024; p = 0.882).

On the other hand, Pearson coefficients for the thinnest point of the cornea were the following: TLR2 expression in neutrophils (r = 0.173; p = 0.287); TLR2 expression in monocytes (r = 0.208; p = 0.198); TLR4 expression in neutrophils (r = −0.219; p = 0.175); and TLR4 expression in monocytes (r = −0.091; p = 0.576).

Finally, there was no association between the elapsed time from the first diagnosis of KC eye and the TLR2 and TLR4 expression in both neutrophils and monocytes (data not shown).

### Correlation between expression of TLR2/TLR4 and proinflammatory molecules

Table 2 shows the correlation between TLR2/TLR4 expression in neutrophils and monocytes with the levels of proinflammatory markers. We found a strong correlation between TLR2 expression in both monocytes and neutrophils (all p < 0.0001) and TLR4 in monocytes (all p < 0.05) with serum levels of IL-1B, IL-6, TNF-α and MMP-9. However, we only found a correlation with MMP-9 serum levels (p = 0.034) for TLR4 expression in neutrophils.

**Table 2:** Correlation between TLR2/TLR4 expression in neutrophils and monocytes with levels of proinflammatory markers.

	**TLR2 neutrophils**	**TLR2 monocytes**	**TLR4 neutrophils**	**TLR4 monocytes**
**IL-1β**	0.695 (p < 0.0001)	0.737 (p < 0.0001)	0.183 (p = 0.161)	0.269 (p = 0.038)
**IL-6**	0.535 (p < 0.0001)	0.614 (p < 0.0001)	0.237 (p = 0.069)	0.328 (p = 0.010)
**TNF-α**	0.583 (p < 0.0001)	0.829 (p < 0.0001)	0.089 (p = 0.501)	0.272 (p = 0.036)
**MMP-9**	0.594 (p < 0.0001)	0.724 (p < 0.0001)	0.275 (p = 0.034)	0.442 (p < 0.0001)

IL = interleukin; KC = keratoconus; MMP-9 = matrix metalloproteinase 9; TLR = Toll-like receptor; TNF-α = Tumor necrosis factor alpha.

**Figure 3:**
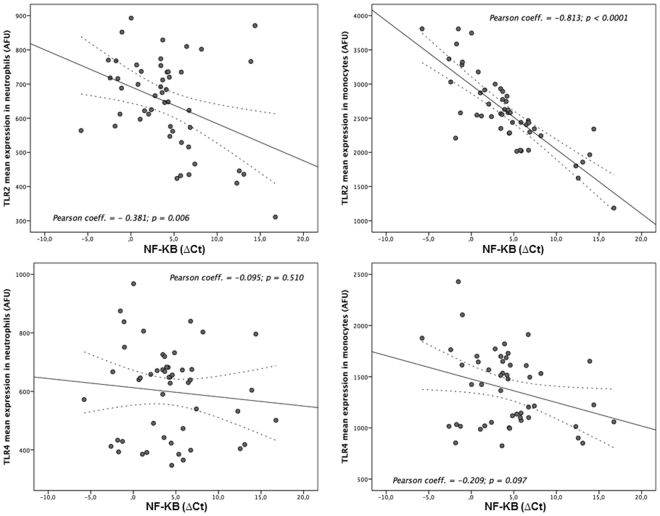
Correlations between NF-kB and TLR2/TLR4 expression levels in neutrophils/monocytes.

Regarding NF-κB, we found a strong correlation with TLR2 expression in neutrophils (r = 0.381; p = 0.006) and monocytes (r = 0.813; p < 0.0001), as well as a weak correlation with TLR4 expression in neutrophils (r = 0.095; p = 0.034) (Fig. 3).

On the other hand, due to KC patients showed more frequent history of allergy disease compared to the control group, we performed a multiple linear regression analysis, adjusted by allergic disease, in order to analyze the independent influence of TLR2/TLR4 expression in monocytes and neutrophils on NF-κB levels, the main proinflammatory factor induced by TLRs. As shown in Table 3, no effects were observed for allergic disease.

**Table 3:** B estimate of TLR2/TLR4 expression in neutrophils and monocytes for NF-kB. Note that due to the collinearity between the TLR2 and TRL4, the influence of each of them on NF-kB expression was assessed in a separate multiple linear regression model.

	**B (CI 95%), p Unadjusted model**	**B (CI 95%), p Adjusted model** ^*^
**TLR2 neutrophils**	200.1 (138.9–261.3), <0.0001	181.8 (119.7–243.7), <0.0001
**TLR2 monocytes**	777.4 (448.4–1106.4), <0.0001	701.7 (362.9–1040.4), <0.0001
**TLR4 neutrophils**	188.1 (117.4–258.9), <0.0001	191.9 (117.6–266.3), <0.0001
**TLR4 monocytes**	559.9 (410.6–709.4), <0.0001	567.8 (410.7–724.9), <0.0001

^*^Adjusted by allergic disease; TLR = Toll-like receptor.

## Discussion

This cross-sectional case-control study has evaluated the expression of TLR2 and TLR4 in monocytes and neutrophils from KC patients compared with control subjects. Remarkably, TLR2 and TLR4 expression in both neutrophils and monocytes was significantly higher in patients with KC compared to control subjects, but the correlation with TLR4 was less robust compared to TLR2. In addition, we found a strong correlation between TLR2 expression in both monocytes and neutrophils and TLR4 in monocytes with serum levels of IL-1B, IL-6, TNF-α, MMP-9 and NF-κB expression. By contrast, no correlation was found between the expression of TLR2 and TLR4 in neutrophils and monocytes with the severity of KC, evaluated by K_2_ and the thinnest point of the cornea.

Despite extensive basic and clinical studies of KC in recent years, the precise mechanisms underlying this pathology still remain unknown. Pathophysiologic components of KC can be largely classified into the following: alterations of the stroma composition, imbalance of pro-inflammatory and anti-inflammatory molecules, imbalance of the enzymes that cause extracellular matrix degradation and their corresponding inhibitors, oxidative stress, and cellular hypersensivity. These events occur simultaneously, but it is yet unclear which precedes the other and which events are necessary for the evolution of the disease^12^. In this regard, several clinical studies of KC support the idea that its pathogenesis may involve an immune and inflammatory component^4–6,12–15^. Interestingly, more recent studies demonstrate that molecular changes associated to KC can be reflected systemically, as it has been shown with oxidative stress^16–19^. These findings are in line with our results, which show that an inflammatory and innate immune response occurs systemically in KC patients. Supporting this claim, there is accumulating evidence that systemic inflammatory changes on oxidative stress may affect the corneal microenvironment in KC^20^. Accordingly, systemic inflammation monitored via the neutrophil-to-lymphocyte ratio was recently associated with progressive KC^19^, and systemic oxidative stress has been also reported in KC patients^17^. Increased frequency of neutrophils indicates proinflammatory conditions, and neutrophils are directly associated with the activation of MMPs, which have been found to be elevated in KC^4,5,19^. Moreover, previous studies by our group and others have demonstrated that inflammatory markers, such as IL-6, TNF-α and MMP-9 are significantly increased in tears of patients with KC^4,5,21–24^. We also observed decreased levels of Lf, IGKC and ZAG in the tears of patients with KC compared to control subjects^6^. On the other hand, it has been described the effects of cyclosporin A in reducing MMP9 and inhibiting inflammation to halt progression of KC^25^. These results suggest that inflammatory and immunological processes may be involved in the pathogenesis of KC, even more when these biomarkers were associated with the severity of the KC^4–6,26^. The results of the present study showed that TLR2 and TLR4 expression in both neutrophils and monocytes was significantly higher in patients with KC compared to control subjects; and that patients with KC also had higher serum levels of IL-1B, IL-6, TNF-α, MMP-9 and NF-κB. In addition, a strong correlation was found between TLR2 expression in both monocytes and neutrophils and TLR4 in monocytes with serum levels of IL-1B, IL-6, TNF-α, MMP-9 and NF-κB expression. Consequently, it is tempting to postulate that such inflammatory response could be a consequence of the TLR activation. In fact, previous studies have shown that the treatment of corneal epithelial cells with neutralizing TLR2- antibodies induced a significant decrease of IL-6, IL-8 and TNF-α levels^11^. Additionally, the preocular tear film contains numerous specific and nonspecific immune components, which include cytokines and cell adhesion molecules^27^; and several cell types, including keratocytes, express IL-6 in response to a stimulation by IL-1β or TNF-α^28^; and TLRs increase secretion of IL-6 and TNF-α in corneal fibroblasts^29^.

Although no association was found between TLR2 and TLR4 expression in neutrophils and monocytes and the severity of KC, the fact that the expression of TLRs is strongly associated with systemic levels of proinflammatory molecules indicates that innate immunity may be involved in the pathophysiology of KC since increased levels of IL-6, TNF-α and MMP-9 in tears were associated with the degree of KC evolution. Moreover, as previously mentioned, systemic inflammation monitored via the neutrophil-to-lymphocyte ratio was recently associated with progressive KC^19^.

This study has some limitations. Firstly, in our study, KC patients and/or control subjects with immunological diseases, including autoimmune diseases, were excluded, and a higher incidence of KC was reported in immune disorders^30,31^. This fact may be excluding potential results on new insights or pathways regarding the association between immune response and KC. However, the inclusion of patients and controls with allergic asthma, atopic dermatitis and allergic rhinitis somehow mitigates this limitation, even more so when the allergy variable was included in the different analyzes performed in the study.

In conclusion, this study reveals that TLR2 and TLR4 in monocytes and neutrophils are overexpressed in KC patients compared to control subjects. Moreover, this overexpression of TLR2/TLR4 is associated to increased serum levels of inflammatory mediators such as NF-kB, IL-6, IL-1B, TNF-α, MMP-9. These results suggest that TLRs may play an important role in the pathophysiology of KC.

## Methods

### Patients

We have designed a cross-sectional, case-control study in which 40 patients with bilateral KC (55% male; mean age, 31.1 ± 10.9 years) and 20 control subjects (55% male; mean age, 30.4 ± 7.6 years), without KC clinical or topographic signs, were enrolled. All KC patients and control subjects were specifically cited for the purposes of this study, and all examinations were performed by the same researcher. Collected data included sex, age, patient’s ocular history, medical history (allergy, eye rubbing), and family history of KC. We have considered allergic conditions the following: allergic asthma, atopic dermatitis, and/or allergic rhinitis. This research was carried out in accordance with the Declaration of Helsinki of the World Medical Association (2008) and approved by the Ethics Committee of Research at Servizo Galego de Saúde. Informed consent was obtained from each patient or control subject after full explanation of the procedures.

Inclusion criteria were the following: 1) Patients with bilateral KC with biomicroscopic signs in both eyes, such as Vogt striae, Fleischer ring, and prominent corneal nerves. Rabinowitz/McDonnell criterion was used for the KC diagnosis^32,33^, and tomographic parameters; 2) Control subjects with normal corneal topography maps and without biomicroscopic signs of KC.

Exclusion criteria included the following: 1) previous surgical intervention in the anterior segment, or past history of corneal trauma or any other corneal pathology; 2) existence of active systemic or ocular inflammation, and current treatment with systemic or local anti-inflammatory drugs; 3) hepatic, renal, hematologic, and immunologic diseases, disorders of thyroid function, uncontrolled diabetes, infections in the days preceding to the sample collection and solid tumors; since they may interfere with the results of the study of molecular markers of innate immunity.

The biomicroscopy exam was performed to detect signs of KC and the corneal topography study was performed to quantify topographic parameters. The modified Rabinowitz-McDonnell test was used to confirm the diagnosis of KC^32,33^, and the severity of KC was classified according to the steepest simulated keratometry reading (K_2_) on the keratometric map (K_2_ < 45 D, mild; K_2_ between 45 and 52 D, moderate; K_2_ > 52 D, severe KC)^4,34^. Quantitative topographic parameters were analysed: steep, flat and average keratometry (K) values, 3 mm irregularity, 5 mm irregularity; and tomographic parameters: posterior elevation and the thinnest point pachymetry of the cornea.

### Ophthalmological Instruments

Basic examination instruments were a Topcon SL-2 biomicroscope, Topcon IS-600 refraction column with trial lenses set, and alphabetic Snellen visual acuity test. As specific examination instruments we used a TOPCON CA-100 topographer (Topcon Medical Systems, Inc., NJ, USA) for corneal topography; and an Orbscan IIz (Orbtek, Utah, USA) for corneal pachymetry and posterior elevation.

### TLR2 and TLR4 expression analysis

TLR2 and 4 expression analyses were performed by flow cytometry in blood samples, withdrawn from all KC patients and control subjects, collected in EDTA-anticoagulated tubes. For the expression analysis of TLR2 and TLR4, monocytes, lymphocytes and neutrophils were separated by their forward and side scattering signal characteristics. Fluorescein isothiocyanate (FITC) anti-TLR2-conjugated monoclonal antibodies (IMMUNOSTEP, Salamanca, Spain) and phycoerythrin (PE) anti-TLR4 conjugated monoclonal antibodies (IMMUNOSTEP, Salamanca, Spain) were used to quantify TLR expression. Samples were analyzed on a FACSAria iiu flow cytometer (BD Biosciences, NJ, USA). Cell fluorescence was measured immediately after staining, and data were analyzed with the use of FACSDiva software (BD Biosciences, NJ, USA). Mean expression of TLR2 and TLR4 in monocytes and neutrophils was expressed as AFU (arbitrary fluorescence units).

### Laboratory Test

Blood samples were collected in chemistry test tubes, centrifuged at 3000 g for 15 minutes, and immediately frozen and stored at −80 °C. IL-1β, IL-6 and TNF-α serum levels were measured by using an immunodiagnostic IMMULITE 1000 System (Siemens Healthcare Global, Los Angeles, CA, USA). On the other hand, serum levels of active MMP-9 (GE Healthcare, Amersham, UK, Little Chalfont, Buckinghamshire, UK) were measured using commercial ELISA kits following manufacturer’s instructions. The intra-assay and inter-assay coefficients of variation (CV) for all molecular markers were <8%. Determinations were performed in a laboratory blinded to clinical and topographic data.

### NF-kB expression analysis

Expression of NF-κB was analyzed by rq-PCR on a Mx3005p system from Stratagene (Thermo Scientific, Waltham, MA, USA). For this analysis, RNA was extracted from 200 μL of EDTA anticoagulated blood sample, with a semiautomatic robot MagnaPure Compact (Roche Diagnostics, Basel, Switzerland). RNA extracts were quantified with a Nanodrop system (Thermo Scientific, Waltham, MA, USA) and cDNA was synthesized for each sample using the High Capacity RNA to cDNA kit (Applied Biosystems, Thermo Scientific, Waltham, MA, USA) according to the provided instructions in a thermocycler T Professional (Biometra GmbH, Göttingen, Germany). rq-PCR was performed with commercially available specific primers and probes for NF-κB (Applied Biosystems, Thermo Scientific, Waltham, MA, USA). NF-κB amplifications were simultaneously performed with a GAPDH amplification, which was used as loading control. Following rq–PCR, quantification of gene expression was made by determining the Ct (for PCR cycle in what amplification it starts, these units are inverse to the NF-κB quantity), based on fluorescence detection, within the geometric region of the semilog view of the amplification plot. Relative quantization of target gene expression was evaluated using the comparative Ct method as previously described by Ross *et al*.^35^. The ΔCt value was determined by subtracting the target Ct of each sample from its respective GAPDH Ct value.

### Statistical analysis

Sample size was calculated by using the statistical EPIDAT 3.1 software (http://www.sergas.es/MostrarContidos_N3_T01.aspx?IdPaxina=62714), considering that TLR2/TLR4 expression in KC patients is >25% compared to control subjects. The minimum calculated sample size was 40 patients with KC and 20 control subjects in order to obtain a statistical power of 80% with a significant difference level of 0.05.

Results were expressed as percentages for categorical variables and as mean (standard deviation) or median and range (25th and 75th percentiles) for the continuous variables depending on whether their distribution was normal or not. The Kolmogorov-Smirnov test was used for testing the normality of the distribution. Proportions were compared using the chi-square or Fisher test, while the continuous variables between groups were compared with the Student’s t or the Mann-Whitney tests depending on whether their distribution was normal or not, respectively. Bivariate correlations were performed using Pearson’s (normally distributed variables) or Spearman (variables without normal distribution) coefficients.

The independent influence of TLR2/TLR4 expression in monocytes and neutrophils on NF-κB levels was assessed by multiple linear regression models. This multivariable linear regression model was adjusted by allergic disease. Results were expressed as Beta estimate with the corresponding 95% confidence intervals (95% CI). A p-value < 0.05 was considered to be statistically significant in all tests. The statistical analysis was conducted in SPSS 20.0 (IBM, Chicago, IL, USA) for Mac.
